# A New Therapeutic Candidate for Cardiovascular Diseases: Berberine

**DOI:** 10.3389/fphar.2021.631100

**Published:** 2021-03-17

**Authors:** Yun Cai, Qiqi Xin, Jinjin Lu, Yu Miao, Qian Lin, Weihong Cong, Keji Chen

**Affiliations:** ^1^ Doctoral Candidate, Dongzhimen Hospital of Beijing University of Chinese Medicine, Beijing, China; ^2^ Laboratory of Cardiovascular Diseases, Xiyuan Hospital of China Academy of Chinese Medical Sciences, Beijing, China; ^3^ National Clinical Research Center for Chinese Medicine Cardiology, Beijing, China; ^4^ Dongfang Hospital of Beijing University of Chinese Medicine, Beijing, China; ^5^ Dongzhimen Hospital of Beijing University of Chinese Medicine, Beijing, China

**Keywords:** berberine, cardiovascular diseases, natural product, therapeutic effects, safety

## Abstract

Cardiovascular diseases (CVD) are the leading cause of death in the world. However, due to the limited effectiveness and potential adverse effects of current treatments, the long-term prognosis of CVD patients is still discouraging. In recent years, several studies have found that berberine (BBR) has broad application prospects in the prevention and treatment of CVD. Due to its effectiveness and safety for gastroenteritis and diarrhea caused by bacterial infections, BBR has been widely used in China and other Asian countries since the middle of the last century. The development of pharmacology also provides evidence for the multi-targets of BBR in treating CVD. Researches on CVD, such as arrhythmia, atherosclerosis, dyslipidemia, hypertension, ischemic heart disease, myocarditis and cardiomyopathy, heart failure, etc., revealed the cardiovascular protective mechanisms of BBR. This review systematically summarizes the pharmacological research progress of BBR in the treatment of CVD in recent years, confirming that BBR is a promising therapeutic option for CVD.

## Introduction

Cardiovascular diseases (CVD) are the leading cause of death in the world, including arrhythmia, atherosclerosis, myocardial infarction, hypertension, hyperlipidemia, myocarditis and cardiomyopathy, and heart failure. These diseases are the results of multiple pathological factors, and their pathogenesis have not been fully elucidated (Mozaffarian et al., 2015; Xu et al., 2018a; Xu et al., 2018b; Timmis et al., 2018). The effects of the current treatment strategies for CVD, including antiplatelet drugs, anticoagulants, angiotensin converting enzyme inhibitors, statins, beta blockers and nitrates, are still limited (Squizzato et al., 2017; Zhao et al., 2017; Blessberger et al., 2018), and the potential adverse effects of some drugs impaired the long-term prognosis and life quality of patients. New drugs discovery and development are constantly being desired.

**FIGURE 1:**
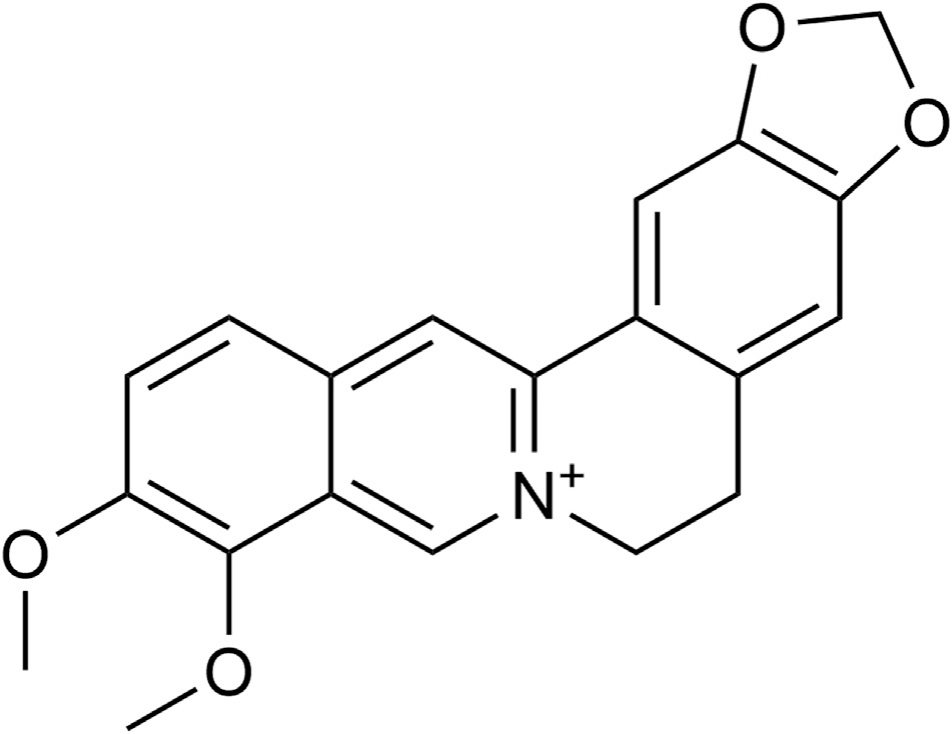
Chemical structure of berberine. Berberine has been found to exhibit biological activities including the reduction of blood sugar, regulation of lipids, as well as anti-arrhythmic and cardio-protective effects. With the progress of modern pharmacological research, the properties of BBR in the treatment of multiple disease have gradually been discovered.

Berberine (BBR), also known as Huang Lian Su, is an isoquinoline alkaloid. The chemical structure of BBR is shown in Figure 1, and its molecular structure is C_20_H_18_NO_4_ and molecular weight is 336.39 g/mol. It is present in the root, rhizome and stem bark of many medicinally important plants, such as *Hydrastis canadensis* (goldenseal), *Coptis chinensis* Franch (Coptis or goldenthread), *Berberis aquifolium* (Oregon grape), *Berberis vulgaris* (barberry), and *Berberis aristata* (tree turmeric) (Chen et al., 2012). The main forms of BBR’s clinical application are hydrochloride and sulfate. In the past, it was mostly used as a kind of antibiotic for intestinal infections. In recent years, BBR has been found to exhibit biological activities including the reduction of blood sugar, regulation of lipids, as well as anti-arrhythmic and cardio-protective effects (Ye et al., 2009). As a natural product-derived drug with development prospects, it has been widely used in China and other Asian countries for its effectiveness and safety (Jin et al., 2016; Ju et al., 2018). With the progress of modern pharmacological research, the properties of BBR in the treatment of multiple diseases have gradually been discovered. Thus, we reviewed the molecular targets and development potential of BBR in the treatment of CVD.

## Arrhythmia

Cardiac arrhythmia is the leading cause of sudden cardiac death. Evidence from a retrospective clinical study showed that BBR (1.2–2.0 g/d, p.o., 12 weeks) had the same efficacy as amiodarone in the treatment of atrial fibrillation (AF), and no adverse effects was observed (Zheng et al., 2017). The anti-arrhythmic effect of BBR was first demonstrated in 1989. The author established an ischemic model by ligating the anterior descending coronary artery in dog. The results showed that BBR could significantly inhibit the occurrence of ischemic ventricular arrhythmias (Huang et al., 1989). In recent years, several studies have further confirmed the antiarrhythmic effect of BBR. To evaluate the effects and mechanisms of BBR on arrhythmia, Cao et al. (2012) established an arrhythmia model by stretching the heart of wistar rats with myocardial infarction. The results showed that BBR inhibited the occurrence of ventricular tachycardia by decreasing the prolongation of repolarization of single-phase action potential and reducing the incidence of ventricular premature beats. Wang et al. (2011) verified the antiarrhythmic effect of BBR (180 mg/kg/d, i.g., 2 weeks) in myocardial infarction model in diabetic rats, and showed such effects may result from BBR regulating inward rectifier K^+^ channels/Kir2.1. Zhou et al. (2015) assessed the effect of BBR on acetylcholine intravenous injection induced AF model in rabbits. The results showed that BBR (1 mg/kg, i.v.) significantly reduced the rate of acetylcholine-induced persistent AF and terminated most of the acetylcholine-induced AF, and BBR (2 mg/kg, i.v.) terminated AF by prolonging the effective refractory period of atrium and the action potential duration of atrial myocytes. The effects may result from BBR prolonging atrial repolarization by blockading the components of the delayed rectifying K^+^ current (Zhou et al., 2015). Hua and Wang, 1994 also confirmed the effect of BBR on K^+^ channels. The results showed that BBR was able to block inward rectifier K^+^ channels and delay rectifier K^+^ channels, and thereby prolong the action potential duration. Furthermore, Li et al. (1995) showed that BBR (10 mg/kg) was able to reduce the incidence of ventricle tachycardia (VT), shorten the VT duration, inhibit Ca^2+^ inwards in at a high Ca^2+^ concentration in reperfusion-induced arrhythmias rats, and the results of *in vitro* experiments showed that BBR (30 μmol/L) promoted Ca^2+^ inwards at a low Ca^2+^ concentration, indicating that BBR may have an effect against reperfusion-induced arrhythmia by inhibiting Ca^2+^ channels. Meanwhile, BBR derivatives such as *p*-chlorobenzyltetrahydroberberine chloride (Cui et al., 2003) and dihydroberberine (Zhang et al., 1997) have also been confirmed to have an effect on arrhythmia. In summary, BBR have effects against ventricular and supraventricular arrhythmias, which might result from inhibiting K^+^ and Ca^2+^ currents.

## Atherosclerosis

Atherosclerosis (AS) is a chronic traumatic disease and the main cause of various cardio-cerebrovascular diseases. Its pathology starts from endometrial damage. The plaque is formed by local deposition of lipids, complex carbohydrates, fibrous tissue, and calcium. The pathogenesis of AS is complex. A current study has found that multiple factors including low density lipoprotein cholesterol (LDL-C) oxidation, platelet adhesion, inflammation, macrophage activation, endothelial cell damage, and smooth muscle cell proliferation are closely related to the development of AS (Spence, 2016). BBR was demonstrated to significantly lower the levels of tumor necrosis factor *α* (TNF-*α*) and interleukin-6 (IL-6) in both serum of apolipoprotein E gene knockout (Apo^-/-^E) mice (5 mg/kg/d, i.v., 12 weeks) and supernatant of human umbilical vein endothelial cells (HUVECs) (50 μmol/L). Furthermore, it decreased the expression of visfatin protein in aorta and down-regulated the protein expression of p38 mitogen-activated protein kinase (p38 MAPK), c-Jun N- terminal kinases (*p*-JNK), and Bax while up-regulated the expression of Bcl-2 in HUVECs. The results of this study indicated that BBR suppressed the inflammatory response and reduced blood lipid levels possibly by inhibiting the p38 MAPK and JNK signaling pathways, which improve endothelial dysfunction and prevent the occurrence of AS (Wan et al., 2018). In a recent study, BBR (78 and 156 mg/kg, p.o., 12 weeks) reduced aortic reactive oxygen species (ROS) production and decreased serum malondialdehyde (MDA), oxidized low-density lipoprotein (ox-LDL) and IL-6 levels in an Apo^−/−^E mice model, demonstrating that BBR may improve endothelial dysfunction by restoring endothelium-dependent vasodilation and attenuating oxidative stress and inflammatory responses, thereby preventing AS (Tan et al., 2020). In addition, an evidence from 16S rRNA sequencing showed that BBR (100 mg/kg, i.g., 13 weeks) significantly altered the community composition structure of the gut microbiota by anti-inflammation and regulating glucose and lipid metabolism (Wu et al., 2020). An *in vitro* experiment reported that BBR (25 µmol/L) was able to inhibit the expression of extracellular matrix metalloproteinase inducer (EMMPRIN) and matrix metalloprotein (MMP)-9 in macrophages stimulated by ox-LDL, which inhibited the progression of AS. The mechanism of action of BBR was possibly through the activation of NF-κB in macrophages, the prevention of IκB-*α* degradation, and the inhibition of p65 in the cytoplasm from entering the nucleus, resulting in MMP-9 and EMMPRIN expression downregulation and AS plaque stabilization (Huang et al., 2012). In-stent restenosis and neoatherosclerosis after percutaneous coronary intervention (PCI) are closely related to inflammatory response. Galectin-3 is an important regulatory factor of inflammation, which is mainly expressed in macrophages. The results of an *in vitro* study demonstrated that BBR (25 µmol/L) might reduce ox-LDL-induced macrophage activation and down-regulate galectin-3 expression by inhibiting the activation of NF-κB and AMPK signaling pathways. However, the clinical results of the same study showed that an additive 300 mg of BBR hydrochloride t.i.d. treatment on top of standard therapy did not further reduce plasma levels of galectin-3 in acute coronary syndrome patients undergoing PCI. This discrepancy between *in vitro* and clinical results is worth being further discussed (Pei et al., 2019). In a rat model, BBR (1–100 µmol/kg, i.v., 4 weeks) combined with atorvastatin calcium reduced the levels of serum total cholesterol (TC), triglyceride (TG), and LDL-C. It also reduced the levels of plasma endothelium ET-1 (ET1) and decreased the expression of lectin-like oxidized low-density lipoprotein receptor-1 (LOX1), which may prevent the transport of LOX1-mediated ox-LDL into macrophages to promote foam cell formation (Chi et al., 2014). BBR (150 mg/kg/d, p.o.) reduced foam cells and macrophage infiltration, decreased TNF-*α* and IL-1*β* levels, upregulated LC3-II protein expression and downregulated P62 protein expression in the aortic tissues in AS rats, indicating that BBR might inhibit plaque formation and attenuated the inflammatory response in aortic tissue by promoting autophagy (Ke et al., 2020). BT_1500_M, a kind of BBR-entrapped micelle, increased BBR deposition in liver and adipose by 107.6 and 172.3%, respectively. BT_1500_M (BBR, 100 mg/kg/day, i.p., 5 months) showed anti-atherosclerotic efficacy in high-fat diet-fed Apo^−/−^E mice *in vivo* and in adipocytes and macrophages AS models *in vitro* (Ma et al., 2020).The development of oxidative stress is also associated with the pathogenesis of AS. BBR can selectively inhibit the expression of gp91phox protein and enhance the activity of superoxide dismutase, thereby reducing the level of superoxide produced by NADPH oxidase in LPS-stimulated macrophages, which may play a role in the prevention and treatment of AS (Sarna et al., 2010).

Neointimal hyperplasia (NH), is the universal response of the vessels to injury which is manifested by abnormal migration and proliferation of vascular smooth muscle cells within the intima, accompanied by deposition of new extracellular matrix. Histologically, NH is similar to AS but consists almost entirely of smooth muscle cells (approximately 20%) and newly synthesized extracellular matrix (ECM) (approximately 80%), and NH is the leading cause of vessel restenosis in both medium term and long term (McMonagle, 2020). With respect to inhibiting the proliferation of smooth muscle cells, a study reported that BBR (10 μmol/L) may inhibit the activation of extracellular signal-regulated kinases (ERKs) by inhibiting the production of intracellular reactive oxygen species (ROS), which inhibits ERK1/2 activation and therefore suppresses the proliferation and migration of vascular smooth muscle cells (VSMC) (Cho et al., 2005). It was confirmed in a rat carotid artery injury model that BBR (100 µg/kg/d, i.v., 4 weeks) may delay or partially inhibit protein kinase B (Akt) phosphorylation, thereby inhibiting the proliferation and migration of VSMCs induced by angiotensin II and heparin-binding epidermal growth factor (Lee et al., 2006). Several recent studies have confirmed the role of BBR in inhibiting VSMC proliferation from several angles. BBR (30 μmol/L) inhibited angiotensin IV-induced VSMC proliferation by activating the PPAR*α*-nitric oxide (NO) signaling pathway (Qiu et al., 2017). BBR (100 µmol/L) may also restrain the migration of human aortic smooth muscle cells by inhibiting the activator protein-1 (AP-1) and NF-κB signaling pathways and decreasing MMP-2/9 and urokinase-type plasminogen activator (u-PA) expression (Liu et al., 2014). BBR (50 µmol/L) inhibited *chlamydia pneumoniae* infection induced VSMC migration by downregulating MMP3 and MMP9 expression via PI3K (Ma et al., 2015). Furthermore, BBR (10 µmol/L) ameliorated pulmonary arterial hypertension (PAH) and vascular remodeling by inhibiting the thioredoxin (Trx)1/*β*-catenin pathway and inhibited hypoxia-induced pulmonary artery smooth muscle cells proliferation, providing a new target toward the pathological mechanism of PAH (Wande et al., 2020). More recent studies have demonstrated that mechanical stretch promoted VSMC proliferation and apoptosis by activating the protein disulfide bond isomerase (PDI) redox system. BBR (100 µmol/L) inhibited the PDI-endoplasmic reticulum (ER) stress system and downregulated caspase-3 and caspase-12 expression, and thereby attenuate the concomitant increase in proliferation and apoptosis of VSMC in response to mechanical stretch (Wang et al., 2020).

As shown above, BBR shows treatment potential for AS and arterial restenosis, and may indirectly prevent and treat AS through certain signaling pathways closely related to AS, such as MAPK, JNK, NF-κB, AMPK, ERK, AKT, PPAR*α*-NO, AP-1, PI3K, Trx1/*β*-catenin, and PDI/MAPK/ERS.

## Hyperlipidemia

The abnormal metabolism of blood lipids, including TC, TG, LDL-C, and high-density lipoprotein cholesterol (HDL-C), is regarded as the main risk factor for the progression of AS and the development of cardio-cerebrovascular diseases. It has been confirmed in animal studies and clinical trials that BBR could reduce blood lipid levels (Wang and Zidichouski, 2018). Proprotein convertase subtilisin/kexin9 type (PCSK9) is an enzyme that mediates LDL receptor (LDLR) degradation. Endotoxin (LPS) can stimulate PCSK9 expression, reduce the level of LDLR protein in liver, and increase TC, TG, and LDL-C levels while decrease HDL-C level, which can be antagonized by BBR through the up-regulation of LDLR mRNA expression. Moreover, BBR (10 or 30 mg/kg/d, i.g., 4 weeks) can down-regulate PCSK9 expression to reduce the plasma concentrations of LDL-C, TG and TC in mice. Therefore, BBR may inhibit LPS-induced dyslipidemia by regulating the PCSK9-LDLR pathway (Xiao et al., 2012). Hepatocyte nuclear factor 4*α* (HNF-4*α*) is a key transcription factor for hepatocyte differentiation, and miR122 is the main microRNA in liver that participates in lipid and glucose metabolism. BBR attenuated TC, TG and LDL-C and increased HDL-C in high-fat diet-induced diabetic mice. In experimental animals, a high dose of BBR (160 mg/kg, i.p.) appeared more effective compared to the lower dose BBR (40 mg/kg, i.p.) in improving the lipid profiles. In addition, the presence of BBR (10 μmol/L) attenuated the elevation of HNF-4*α* protein and miR122 expression in palmitate (PA)-incubated HepG2 cells. The above data indicated that BBR treatment could reduce the dysregulation of gluconeogenesis and lipid metabolism changes in diabetic mice and PA-induced HepG2 cells by regulating the HNF-4*α*-modulated miR122 pathway (Wei S et al. (2016). Olanzapine (OLZ), a second-generation antipsychotic drug, has adverse effects such as dyslipidemia and insulin resistance mediated by the regulation of the AMP-activated protein kinase-*α* (AMPK*α*)-sterol regulatory element binding protein (SREBP) pathway, which also increases the risk of CVD. However, in an OLZ-induced adipogenesis model established in 3T3-L1 adipocytes, it was confirmed that BBR (5 µmol/L) may reverse the upregulation of SREBP and the downregulation of AMPKα phosphorylation to prevent OLZ-induced lipid metabolism disorders (Li et al., 2016). In clinical trials, a pre-mixed nutraceutical combination approved in Italy, consisting of 500 mg BBR, 200 mg red yeast rice and 10 mg policosanols, showed a cholesterol-lowering effect. It significantly reduced TC and LDL-C levels in high cholesterol patients (Affuso et al., 2010) and in elderly patients with high cholesterol who were intolerant to statins (Marazzi et al., 2011). In a parallel controlled study, a combination of BBR with policosanol, red yeast extract, folic acid and astaxanthin, and BBR alone were found to have similar efficacy and both could reduce TC, TG and LDL-C levels to different degrees (Cicero et al., 2007). Some meta-analyses have also confirmed that BBR or BBR-containing health products have beneficial effects on TC, LDL-C and HDL regulation with good safety (Lan et al., 2015; Wei X et al., 2016; Pirro et al., 2016), showing similar cholesterol-lowering effects of statins (Weng et al., 2010). In a recent double-blind, randomized, placebo-controlled dose-ranging study, an ionic salt berberine ursodeoxycholic acid (BUDCA) formed between BBR and ursodeoxycholic acid significantly reduced serum concentrations of TC and LDL-C, but not TG and HDL-C concentrations, with the maximum dose of 2,000 mg/d at day 28. BUDCA shown to be well tolerated, and no significant adverse effects were reported even at doses of 2,000 mg/d (Di Bisceglie et al., 2020). In summary, the lipid-lowering effect of BBR may be related to its regulating effects on PCSK9-LDLR, HNF-4*α*-miR122, and AMPK*α*-SREBP pathways. However, although BBR is proved to reduce the levels of TC, TG, and LDL-C, its effect on HDL has not been confirmed, and hence, clinical administration of BBR as a lipid-lowering drug needs to be further explored.

## Hypertension

As a chronic disease, hypertension is a major risk factor for increased mortality in patients with CVD. The control of blood pressure is related to the reduction of the occurrence of complications such as stroke, myocardial infarction, heart failure, AS, and ventricular arrhythmia (Ventura and Lavie, 2019). A meta-analysis showed that in the treatment of hypertension, lifestyle intervention combined with BBR reduces blood pressure better than lifestyle intervention or placebo alone, and BBR combined with oral antihypertensive drug also reduced blood pressure better than the same antihypertensive drug. The usual intake of BBR was 0.6–2.7 g per day. Additionally, it is noteworthy that no serious adverse effects were reported in 27 randomized controlled clinical trials with BBR treatment alone (Lan et al., 2015). In a 2 years clinical study that analyzed biochemical markers of renal function and color Doppler ultrasound imaging, BBR showed a renal protective effect for hypertensive patients with type-2 diabetes. In addition to baseline treatment, patients in the add-on group received oral BBR for 24 months. The dose of BBR was 0.1 g three times per day, with a 2 weeks no-treatment intervals every 5 months. The associated mechanism may be the suppression of inflammation and oxidative stress in patients with hypertension and diabetes, thereby improving renal hemodynamics and curtailing kidney injury (Dai et al., 2015). Nitric oxide (NO) can promote vasodilation, prevent vasodilation disorders, and protect the vascular endothelium. In a recent clinical study, trimetazidine combined with BBR was administered to coronary heart disease patients with comorbid essential hypertension for the first time. It was found that trimetazidine combined with BBR could significantly enhance the expression of endothelial NO synthase gene (eNOS), increase the expression of NO, and improve flow-mediated dilation, indicating the improved efficacy of combined drugs (Zhang et al., 2018). In a recent animal experiment, BBR (50 mg/kg/d) was used as an intervention in spontaneously hypertensive rats (SHR). The findings showed that blood pressure and circulating endothelial microparticles level were partly reduced. In addition, BBR maintained arterial elasticity by reducing aortic pulse wave velocity and increasing the content of arterial media elastin fiber, indicating that endothelial function was improved by maintaining better endothelium-dependent vasodilation. (Zhang et al., 2020). The contraction of vascular smooth muscle cells (VSMC) is the main cause of vascular stiffness, mainly influenced by the regulation of calmodulin (CaM)-dependent myosin light chain (MLC). Increased Ca^2+^ influx causes intracellular Ca^2+^ concentration to rise momentarily, which leads to MLC phosphorylation and causes VSMC contraction (Ratz et al., 2005). Transient receptor potential vanilloid 4 (TRPV4) is a Ca^2+^ permeable cation channel (Ueda et al., 2011). It was reported that BBR (100 mg/kg/d, p.o., 7 days) reduced blood pressure in deoxycorticosterone acetate-induced hypertensive mice and the dose of 50 mg/kg/d alleviated vascular stiffness in elderly Apo^−/−^E mice. Such results were possibly mediated by the reduction of Ca^2+^ levels and CaM/MLC activity by BBR through the inhibition of the TRPV4 channel, which caused VSMC to relax and induced the anti-hypertensive as well as anti-vascular aging effects (Wang et al., 2015a). The progression of hypertension usually involves damage to the vascular endothelial cells, accompanied by inflammation and apoptosis (Chung et al., 2004). The activation of myeloid differentiation factor 88 (MyD88)-dependent toll-like receptor 4 (TLR4) signaling pathway can promote the expression of interleukin-1 receptor-associated kinase, nuclear factor-κB (NF-κB), and AP-1, and thus lead to the production of a large number of inflammatory factors, such as cyclooxygenase-2 (COX-2), IL-1 and IL-6, which are associated with hypertension-induced endothelial damage. BBR (1.25, 2.5, and 5 µmol/L) could inhibit the apoptosis of aortic endothelial cells isolated from SHR, decrease the expression of TLR4, MyD88, NF-κB, IL-6 and TNF-*α*, and have a certain protective effect on hypertension-induced vascular endothelial injury (Romero et al., 2011; Wang and Ding, 2015). CXC chemokine receptor 4 (CXCR4) plays a key role in the mediation of EPCs for endothelial repair (Sainz and Sata, 2007). In a recent study, EPCs were isolated from prehypertensive patients, cultured, and transplanted into the nude mice model of carotid artery injury. In the BBR (5 μmol/L) pretreated group, EPC significantly accelerated *in vivo* re-endothelialization and decreased the expression of CXCR4 and downstream Janus kinase-2 (JAK-2), indicating that BBR might protect endothelial cells by interfering with the CXCR4/JAK-2 signaling pathway (Shao et al., 2018). ER stress is closely related to the occurrence of hypertension and ROS is one of the major mediators (Schröder and Kaufman, 2005). BBR (1 *μ*mol/L) may inhibit endothelium-dependent contractions by activating the AMPK pathway, thereby inhibiting ER stress, eliminating ROS, and consequently downregulating COX-2expression in carotid arteries of SHR, which shows a protective effect on vascular function (Liu et al., 2015). In short, BBR may reduce blood pressure and prevent arteriosclerosis and endothelial damage caused by hypertension by regulating the TRPV4, MyD88-TLR4, CXCR4/JAK-2 and AMPK pathways.

## Ischemic Heart Disease

Ischemic heart disease (IHD) is a major contributor to the global disease burden, and remains a substantial public health challenge worldwide (Dai et al., 2020a; Virani et al., 2020). Myocardial infarction (MI) and ischemia-reperfusion injury (IR)-induced myocardial cell death are the main cause of increased IHD morbidity and mortality (Perricone and Heide, 2014). MI is the death of myocardial cells caused by local ischemia, while timely myocardial reperfusion through thrombolytic or PCI restores blood flow in the occluded coronary artery. However, this process will inevitably cause further injury to myocardia (Hashmi and Al-Salam, 2015). Oxidative stress, Ca^2+^ overload, inflammatory response, and apoptosis are key factors in the ischemic process (Hausenloy and Yellon, 2013; Perricone and Heide, 2014). Results of many animal experiments have confirmed that BBR has a protective effect on myocardial ischemia/reperfusion (MI/R) injury. In an experimental study, after being pre-administrated into rat models of ischemia *in vivo*, BBR (100 mg/kg/d, i.g., 2 weeks) was found to significantly reduce the infarct area during IR injury, improve cardiac function, and reduce the concentration of AMPK, the ADP/ATP ratio and the AMP/ATP ratio in myocardial ischemic areas. Therefore, BBR may regulate AMPK activity in non-ischemic and ischemic areas of the heart (Chang et al., 2012). Despite the improvement of coronary ischemia after myocardial reperfusion, a large number of MI patients suffer from insufficient myocardial perfusion due to impaired microvascular function. The induction of angiogenesis can reduce the progression of myocardial infarction and improve cardiac function (Van der Laan et al., 2009). A recent study reported that BBR (100 μmol/L) can increase miR-29b expression, activate Akt in endothelial cells, and promote endothelial cell proliferation and migration, which confirmed the importance of miR-29b in ischemic myocardial remodeling (Zhu et al., 2017). Diabetes increases the risk of ischemic heart disease. BBR (200 mg/kg/d, i.g., 12 weeks) improved cardiac functional recovery in diabetic rats subjected to I/R (Chen et al., 2014). It has also been reported that BBR (200 mg/kg/d, i.g., 2 weeks) may reduce oxidative stress and myocardial inflammation by activating the silent information regulator 1 (SIRT1) signaling pathway (Yu et al., 2016), reduce ER stress-induced apoptosis by activating the AK2/signal transducer and activator of transcription (STAT)3 signaling pathway (Zhao et al., 2016), reduce cardiomyocyte apoptosis by improving mitochondrial dysfunction (Wang et al., 2015b), and protected the myocardial injury via the HIF-1a/BNIP3 pathway (Zhu et al., 2020) in MI/R injury rats. *In vitro* experiments revealed the mechanism of the protective effect of BBR on MI/R injury might be it (50 μmol/L) inhibited apoptosis by activating the AMPK, PI3K-Akt-eNOS, Smad7, and Notch1/Hes1-PTEN/Akt signaling pathways in cardiomyocytes (Chen et al., 2014; Yu et al., 2015; Yao et al., 2018). In addition, BBR (50 μmol/L) may protect myocytes by reducing the expression of caspase-3 (Yao et al., 2018), inhibiting autophagy activation (Huang et al., 2015), promoting mitochondrial autophagy, and promoting cardiomyocyte proliferation (Zhu et al., 2020). When BBR (10 mg/kg/d, i.g., 5 days) extract was combined with high-intensity interval training (HIIT) in a rat model of myocardial IR injury, the transcript levels of vascular endothelial cell growth factor, fibroblast growth factor-2, and platelet response factor-1 were significantly increased. In addition, caspase-3 protein level and infarct size were significantly reduced after 7 days of reperfusion. It can be suggested that HIIT and BBR, alone or in combination, might exert an effect on reducing myocardial infarct size by reducing caspase-3 protein expression and increasing the expression of angiogenesis-promoting factors (Banaei et al., 2020). In summary, BBR may reduce apoptosis and improve myocardial I/R injury by regulating the expression and/or activity of the molecular factors including AMPK, miR-29b, PI3K-Akt-eNOS, SIRT1, Notch1/Hes1-PTEN/Akt, AK2/STAT3, HIF-1a/BNIP3, and Smad7.

## Myocarditis and Cardiomyopathy

Myocarditis is an inflammation of the myocardium caused by infectious, idiopathic or autoimmune causes. A wide spectrum of infectious pathogens including viruses, bacteria, and chlamydia, as well as toxicity and hypersensitivity reactions may cause cardiac damage and lead to inflammation. Viruses (eg, enteroviruses such as coxsackievirus) are the most common infectious pathogens reported in acute myocarditis. Viral proliferation in cardiomyocytes can cause tissue damage, and viral entry through its receptor activates the immune signaling systems, which contributes to cytoskeletal remodeling of host cells. In addition, ongoing inflammation results in changes in cardiac structure and the decline in function, which leads to the development of dilated cardiomyopathy (Sagar et al., 2012). Currently, coxsackievirus B3 (CVB3)-induced acute myocarditis mice model is regarded as the primary animal model for myocarditis, which shows many similarities to clinical myocarditis (Huber, 2016). A recent study replicated CVB3 virus infection in human cervical carcinoma (HeLa) cells or primary cardiomyocyte models *in vitro* and demonstrated that BBR (100 μmol/L) inhibited the phosphorylation levels of JNK and p38 MAPK in cells, thereby inhibiting the replication of CVB3 *in vitro* (Dai et al., 2017). To explore the antiviral effects of BBR *in vivo*, mouse model of CVB3-induced myocarditis was established with intraperitoneal infection, and the results suggested that BBR (100 mg/kg, i.g., 7 days) might reduce cardiac injury and myocardial viral titer, and improve the survival rate and cardiac function as well. In addition, BBR treatment inhibited macrophage infiltration and production of pro-inflammatory cytokines/chemokines such as TNF-*α*, IL-6, IL-1*β*, CCL2, CCL5, and CXCL10 in mice of CVB3-induced myocarditis by inhibiting CVB3 replication. These results demonstrated the beneficial effects of BBR in alleviating CVB3-induced myocarditis *in vivo* (Dai et al., 2020b). Th17 and Th1 are important immune cells involved in the pathogenesis of myocarditis, producing pathogenic cytokines such as IL-17 and IFNg, respectively, which facilitate the development of such autoimmune disease (Tajiri et al., 2012). A recent study using purified porcine cardiac myosin-induced experimental autoimmune myocarditis (EAM) model in Lewis rats indicated that BBR treatment (200 mg/kg/d, i.g., 3 weeks) inhibited Th17/Th1 cell subpopulation differentiation, significantly reduced left ventricular dysfunction and reversed disease progression in EAM rats. Moreover, BBR significantly inhibited the overexpression of phosphorylated (p)-STAT1, STAT3 and STAT4. Thus, BBR could improve EAM by differentially regulating the activities of p-STAT1, p-STAT3 and p-STAT4, which further inhibited the differentiation of Th17 and Th1 cells (Liu et al., 2016). Dilated cardiomyopathy is the most common form of cardiomyopathy induced by a variety of causes, ranging from myocarditis to alcohol and other toxins such as drugs, cardiotoxins and chemotherapeutic agents, to rheumatic, endocrine and metabolic diseases, all of which can lead to reduced left ventricular systolic function and left ventricular or biventricular dilatation (Hänselmann et al., 2020). The anthracycline anticancer drug doxorubicin (DOX) is an effective and commonly used chemotherapeutic agent for the treatment of malignancies, and its main side effect is cardiotoxicity. The main mechanisms are increased oxidative stress, down-regulation of gene expression and induction of apoptosis (Chatterjee et al., 2010). Sirtuin 3 (Sirt3) is the major mitochondrial deacetylase, and to date, studies of Sirt3 have been limited to cell culture systems (Lombard et al., 2007). Sirt3 overexpression contributes to the reduction of DOX cytotoxicity in H9c2 cardiomyocytes. In a pretreated H9c2 cell model, BBR (1 and 10 μmol/L) increased Sirt3 protein levels in the presence of DOX and inhibited DOX-induced caspase 9 and 3-like activation (Coelho et al., 2017). In a recent study a rat model of DOX-induced cardiotoxicity was established. Compared with the DOX group, serum creatine kinase (CK), creatine kinase isoenzyme (CK-MB) and MDA levels were decreased and superoxide dismutase (SOD) and catalase (CAT) levels were increased in the BBR-treated group. BBR (10 mg/kg/d, i.g., 10 days) attenuated mitochondrial Ca^2+^ overload, and restored the DOX-induced loss of mitochondrial membrane potential. BBR showed a protective effect against DOX-induced free radical damage in cardiac tissue, possibly by inhibiting intracellular Ca^2+^ elevation and attenuating mitochondrial dysfunction (Xiong et al., 2018). Furthermore, a study, intervening in a DOX-induced cardiac injury rat model with BBR (20 mg/kg, i.g., 10 days) and in a DOX-induced H9c2 cell injury model with 1 μmol/L BBR, demonstrated BBR’s protective effect against DOX-induced cardiovascular injury, which might be associated with upregulation of SIRT1 and downregulation of p66shc expression, resulting in inhibition of ROS production, apoptosis and mitochondrial damage, thus improving cardiac dysfunction (Wu et al., 2019). Diabetic cardiomyopathy (DCM) is a disease in which there is abnormal myocardial structure and function in the absence of other cardiac risk factors (Jia et al., 2018). In a high-sucrose and high-fat diet-fed and streptozotocin-induced rat model of DCM, BBR (10, 30 mg/kg/d, i.g., 16 weeks) significantly improved cardiac diastolic and systolic function, as well as preventing cardiac hypertrophy. BBR intervention partially reversed the metabolic disorders of biomarkers including phosphatidylcholine, phosphatidylethanolamine and sphingolipids (Dong et al., 2018). BBR treatment (100 mg/kg/d, i.g., 16 weeks) in diabetic rats partially improved cardiac function and attenuated the development of cardiac fibrosis in rats with DCM, probably by increasing cardiac AMPK and AKT activities and inhibiting glycogen synthase kinase-3 activity, as confirmed in a palmitate-induced hypertrophy model of H9C2 cell. In addition, the hypertrophy of H9C2 cells was inhibited by upregulating the expression of α-myosin heavy chain and downregulating the expression of *β*-myosin heavy chain (Chang et al., 2015). In General, BBR may attenuate myocardial injury by regulating the activity of JNK-p38 MAPK, SIRT1-p66shc, AMPK-AKT, GSK3β, STAT1, STAT3, STAT4, Sirt3, and other proteins.

## Heart Failure

Heart failure (HF) is the end stage of multiple heart diseases. Ventricular remodeling is an important strategy for the treatment of HF (Konstam et al., 2011). As early as 1988, a clinical study reported the protective effect of BBR on 12 patients with refractory congestive heart failure. The study reported that intravenous infusion of BBR at a dose of 0.2 mg/kg per minute could reduce the end-diastolic pressure of the right atrium and left ventricle, increase the left ventricular ejection fraction, decrease the arteriovenous oxygen difference, and have a significant effect on hemodynamics (Marin-Neto et al., 1988). Moreover, oral administration of BBR is also proved effective for HF. A total of 156 patients with chronic heart failure (CHF) were randomly divided into a treatment group and a placebo group. In the treatment group, BBR at a dose of 1.2 g/d was orally administered in addition to conventional treatment. Compared with the control group, the BBR group showed an increase in the 6 min walking distance and left ventricular ejection fraction, improvement of the dyspnea-fatigue index, a decrease in the frequency and complexity of ventricular premature contractions (VPCs), indicating that BBR might improve quality of life and reduce VPCs and mortality in CHF patients. (Zeng et al., 2003). In animal experiments, different doses of BBR have been used to treat diastolic heart failure in rats, and could reduce the left ventricular end-diastolic pressure and decrease the Ca^2+^ levels in myocardial cells in a dose-dependent manner (Zhang et al., 2008). It has already been reported that the advanced progression of pathologic myocardial hypertrophy can lead to pressure overload-induced cardiac dysfunction or HF (Frey and Olson, 2003). Furthermore, the autophagic process that causes the degradation of organelles and proteins is closely related to the pathogenesis of HF, which can remove the damaged proteins and organelles, especially mitochondria and ER (Nakai et al., 2007; Gottlieb and Mentzer, 2010). Studies have shown that by inhibiting the activity of the mammalian target of rapamycin (mTOR) and the phosphorylation of its upstream extracellular signal-regulated kinase (ERK1/2) and p38 mitogen-activated protein kinase (MAPK), BBR (10 mg/kg/d, p.o., 4 weeks) could enhance autophagy and inhibit ER stress, thus playing an important role in preventing pressure overload-induced myocardial hypertrophy and apoptosis (Li et al., 2014; Hashemzaei et al., 2017; Chen et al., 2020). Additionally, BBR also improved cardiac dysfunction and myocardial hypertrophy by upregulating putative kinase 1 (PINK1)/cytosolic E3 ubiquitin ligase (Parkin)-mediated mitochondrial autophagy and inhibited cardiomyocyte apoptosis and mitochondrial damage (Abudureyimu et al., 2020). It has also been reported that through the P21-activated kinase 1 (Pak1)-dependent signaling pathway, BBR (3 mg/kg/d) might suppress the upregulation of Smad3-mediated muscle-specific F-box protein (Fbxo32) in downstream promoter region and take part in the inhibition of myocardial hypertrophy (Tsui et al., 2015). In brief, BBR may improve cardiac function and prevent heart failure by regulating the mTOR pathway, the Pak1 pathway and Ca^2+^ concentration.

## Safety

In 2015, a meta-analysis including 27 RCTs on BBR reported that no serious adverse effect was found during the treatment of type 2 diabetes, hyperlipidemia, and hypertension and the treatments with BBR in such trials were at a relatively low cost compared with other first-line medicines and treatments (Lan et al., 2015). However, human pharmacokinetic data show that BBR is poorly absorbed from the intestinal tract and rapidly metabolized *in vivo*, resulting in its low oral bioavailability. Whereas, increasing gastrointestinal reactions were found when treated with large dosages of BBR, which is possibly a major obstacle to the application of BBR (Wang et al., 2017). With regard this, a transdermal formulation of BBR and the BBR precursor dihydroberberine were developed (Buchanan et al., 2018). Transdermal administration of BBR increased circulating levels of BBR and avoided side effects on the gastrointestinal, kidney and liver. A nano-system BT1500M encapsulated with BBR in the latest studies can increase gut absorption and intracellular uptake of BBR which is thought to be promising in clinical applications (Ma et al., 2020).

## Summary and Outlook

**FIGURE 2:**
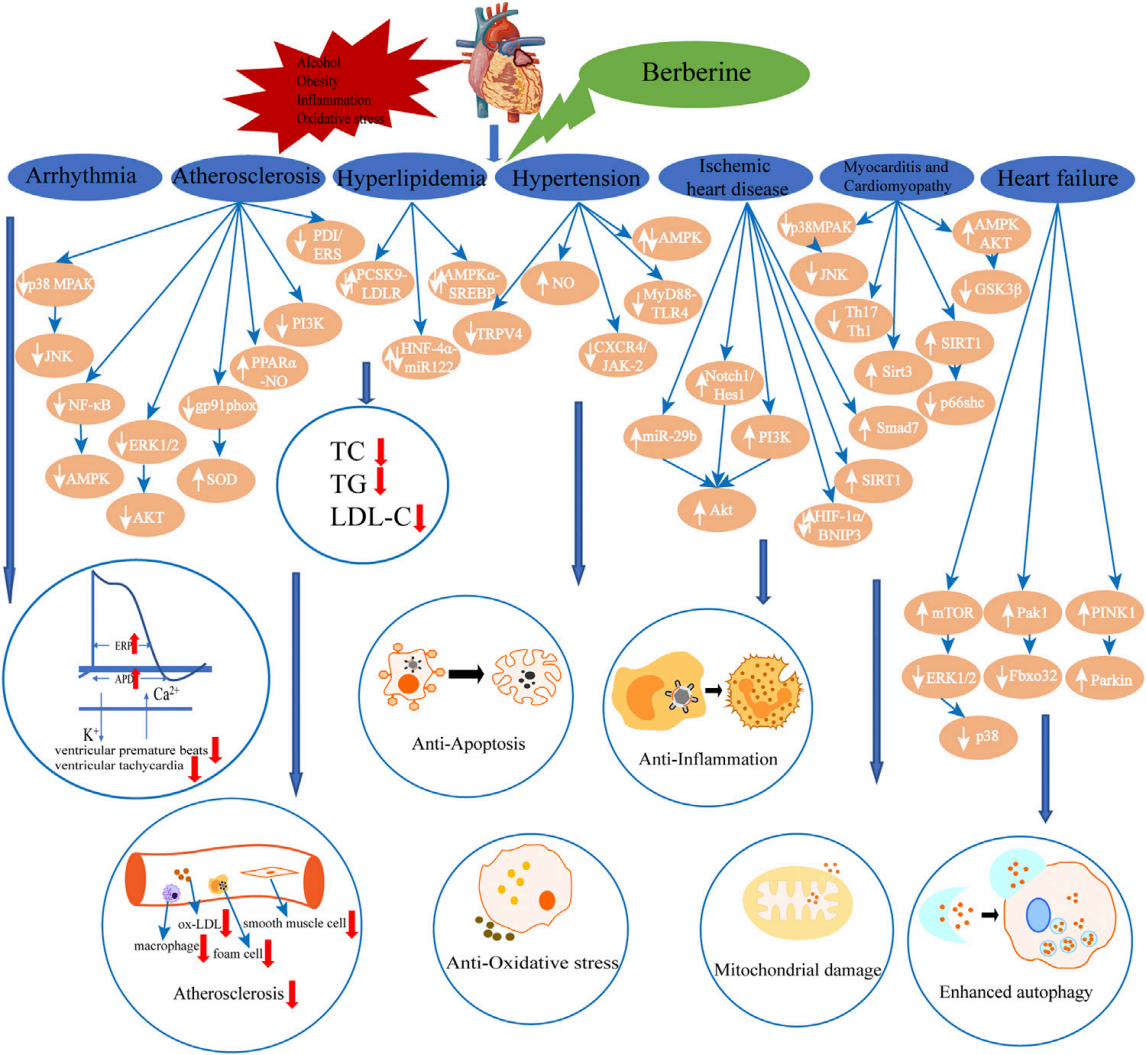
The summarization of effects and main signal pathways regulated by berberine in its organ-and tissue-protective effects from both in vivo and in vitri studies. (↑: increase; ↓: decrease).

Previous experimental and clinical studies of BBR in the treatment of CVD were relatively comprehensively reviewed which indicated the therapeutic effects of BBR on various CVD, including arrhythmia, atherosclerosis, hyperlipidemia, hypertension, ischemic heart disease, myocarditis and cardiomyopathy and heart failure. Such effects are mediated via multiple targets, including pathway targets such as MAPK, JNK, NF-κB, AMPK, ERK, AKT, TRPV4, MyD88-TLR4, and so on (Figure 2, Table 1). A recent review summarizes the pharmacological properties and therapeutic applications of BBR in CVD and metabolic diseases, including cardiac hypertrophy, HF, AS, IR, stroke, arrhythmia, DM, and non-alcoholic fatty liver disease, through 2019. It provided multiple perspectives on the efficacies and mechanisms of BBR interventions in a wide range of diseases, giving a glimpse of the therapeutic potential of BBR (Feng et al., 2019). Furthermore, BBR also has advantages in cancer, digestive and neurological diseases therapy (Song et al., 2020). The present review summarized the main pharmacological effects of BBR on CVD and related mechanisms, including the up-to-date work on myocarditis and cardiomyopathy, which provided more reference for the future development of BBR drugs. In addition, the effects of BBR on protecting vascular endothelium, reducing the area of myocardial infarction and improving heart function undoubtedly confirm the clinical prospects of BBR for improving long-term cardiac prognosis. A recent review described that BBR exerts antioxidant, hepatoprotection, neuroprotection, nephroprotection, pulmonary protection, as well as immunomodulatory, antidiabetic and antitumor effects by activating the nuclear factor erythroid 2 - related factor 2 (Nrf2) signaling pathway, which provides further insights into the treatment of BBR for CVD although more studies are still needed to demonstratae the modulatory effects of BBR (Ashrafizadeh et al., 2020). In general, BBR is a promising option for CVD. The main formulation of BBR in clinical application is its hydrochloride or sulfate (Jin et al., 2016; Lin and Zhang, 2018), which have low oral bioavailability. Modern oral formulations, BBR with different types of nanocarriers, overcome this shortcoming (Mirhadi et al., 2018), and reduce the frequency of administration as much as possible, which not only increases patient compliance, but also reduces potential side effects. However, since most of the mechanisms of actions are obtained from laboratory, it is necessary to get more conclusion from clinical trials to better evaluate the safety and effectiveness of BBR, and to further clarify its mechanisms.

**TABLE 1:** Summary of the mechanisms of action of berberine in the treatment of CVD.

Disease	Mechanisms
Arrhythmia	Inhibits the activation of K^+^ current → Prolongs the effective refractory period of the atrium and the action potential duration of the atrial myocytes → Reduces the incidence of ventricular premature beats and inhibit the occurrence of VT^a^ Hua and Wang (1994), Wang et al. (2011), Cao et al. (2012), Zhou et al. (2015). Inhibits Ca^2+^ current → Reduces the incidence of VT and shorten the duration of VT →Treats reperfusion arrhythmia Li et al. (1995).
Atherosclerosis	Inhibits the p38 MAPK and JNK signaling pathways → Inhibits the inflammatory response → Reduces blood lipids → Improves endothelial dysfunction → Prevents AS^b^ Wan et al. (2018) Inhibits the ROS^c^ production and reduces MDA^d^, ox-LDL^e^ and IL-6^f^ levels →Restores the endothelium-dependent vasodilation → Reduces the oxidative stress and inflammatory response → Improves endothelial dysfunction → Prevents AS Tan et al. (2020) . Inhibits the NF-κB and AMPK signaling pathways → Reduces macrophage activation → Inhibits foam cell formation → Reduces the levels of TC^g^, TG^h^, and LDL-C^i^ Huang et al. (2012), Chi et al. (2014), Pei et al. (2019), Ke et al. (2020). Inhibits the expression of gp91phox protein and enhance the activity of SOD^j^ → Reduces the superoxide levels → Inhibits oxidative stress → Prevents AS Sarna et al., (2010). Inhibits the activation of ERK1/2 and the phosphorylation of AKT → Inhibits smooth muscle cell proliferation and migration → Prevents AS and arterial restenosis Cho et al. (2005), Lee et al. (2006). Activates the PPARα-NO^k^ signaling pathway/Inhibitis the PI3K signaling pathway/Inhibits the PDI/MAPK/ERS system → Inhibits VSMC^l^ proliferation Ma et al. (2015), Qiu et al. (2017), Wang et al. (2020).
Hyperlipidemia	Regulates the PCSK9-LDLR pathway → Reduces the level of LDLR protein in the liver → Reduces the plasma concentrations of LDL-C, TG, and TC Xiao et al. (2012) Regulates the HNF-4α-miR122 pathway → Reduces gluconeogenesis and lipid metabolism changes in the liver Wei S et al. (2016). Regulates the AMPKα-SREBP pathway → Prevents olanzapine-induced lipid metabolism disorders Li et al. (2016).
Hypertension	Increases the expression of NO → Promotes vasodilation → Maintains arterial elasticity and improves endothelial function Zhang et al. (2018), Zhang et al. (2020). Inhibits the TRPV4 channels → Relaxes the vascular smooth muscle → Treats hypertension and vascular aging Ueda et al. (2011), Wang et al. (2015a). Inhibits the MyD88-TLR4 pathway → Inhibits endothelial apoptosis → Protects the vascular endothelium from damage Chung et al. (2004), Romero et al. (2011), Wang and Ding (2015). Inhibits the CXCR4/JAK-2 signaling pathway → Protects endothelial cells Sainz and Sata (2007), Shao et al. (2018). Activates the AMPK pathway → Inhibits endoplasmic reticulum stress in endothelial cells → Protects vascular function Schröder and Kaufman (2005) Liu et al. (2015).
Ischemic heart disease	Regulates the activity of AMPK in non-ischemic and ischemic regions of the heart → Reduces infarct areas during IR^m^ injury → Improves cardiac function Chang et al. (2012) Increases the expression of miR-29b → Activates Akt in endothelial cells → Promotes the proliferation and migration of endothelial cells → Improves myocardial remodeling Van der Laan et al. (2009), Zhu et al. (2017) Activates the AMPK, PI3K-Akt-eNOS, Notch1/Hes1-PTEN/Akt, AK2/STAT3, and Smad7 pathways → Reduces apoptosis → Reduces MI/R^n^ injury Chen et al. (2014), Yu et al. (2015), Zhao et al. (2016), Yao et al. (2018) Activates the SIRT1 pathway → Reduces oxidative stress and cardiac inflammation → Reduces MI/R injury Yu et al. (2016) Regulates the HIF-1α/BNIP3 pathway → Promotes mitochondrial autophagy, promotes cardiomyocyte proliferation, and inhibits cardiomyocyte apoptosis → Reduces MI/R injury Zhu et al. (2020). Inhibits caspase-3 protein expression → Increases VEGF^o^, FGF2^p^ and TSP-1^q^ expression → Reduces myocardial infarct size Banaei et al. (2020).
Myocarditis and cardiomyopathy	Inhibits the p38 MAPK and JNK pathway → Inhibits CVB3^r^ replication → Inhibits macrophage infiltration and pro-inflammatory factors production → Reduces cardiac injury Dai et al. (2017), Dai et al. (2020b) Inhibits the Th17/Th1 cell differentiation → Improves left ventricular function → Improves EAM^s^ Liu et al. (2016) Increases the Sirt3 protein levels → Inhibits caspase 9 and 3-like activation → Attenuates cardiotoxicity Coelho et al. (2017) Inhibits the elevation of intracellular Ca^2+^ → Alleviates mitochondrial dysfunction → Decreases CK^t^, CK-MB^u^ and MDA levels and increases SOD and CAT^v^ levels → Improves cardiac dysfunction Xiong et al. (2018) Upregulates of SIRT1 and downregulates the p66shc expression → Inhibits the ROS production, apoptosis and mitochondrial damage → Improves cardiac dysfunction Wu et al., (2019). Increases the cardiac AMPK and AKT activity and inhibits GSK3β activity → Inhibits cardiac fibrosis → improves cardiac function Chang et al. (2015).
Heart failure	Reduces the end-diastolic pressure of the right atrium and left ventricle, increases the left ventricular ejection fraction, and decreases the arteriovenous oxygen difference → Reduces the incidence of ventricular arrhythmias → Improves quality of life Marin-Neto et al. (1988), Zeng et al. (2003) Decreases the Ca^2+^ levels in myocardial cells → Decreases the left ventricular end-diastolic pressure Zhang et al. (2008) Regulates the mTOR pathway → Inhibits the phosphorylation of ERK1/2 and p38 → Enhances autophagy and inhibits endoplasmic reticulum stress → Prevents myocardial hypertrophy and apoptosis Li et al. (2014), Hashemzaei et al. (2017) Activates the Pak1 pathway → Inhibits the upregulation of Fbxo32 → Treats myocardial hypertrophy Tsui et al. (2015) Upregulates the PINK1/Parkin-mediated mitochondrial autophagy → Inhibits the cardiomyocyte apoptosis and mitochondrial damage → Improves the cardiac dysfunction and myocardial hypertrophy Abudureyimu et al. (2020).

a, ventricular tachycardia; b, Atherosclerosis; c, reactive oxygen species; d, malondialdehyde; e, oxidized low-density lipoprotein; f, interleukin-6; g, Total cholesterol; h, Triglyceride; i, LDL cholesterol; j, superoxide dismutase; k, Nitric oxide; l, vascular smooth muscle cells; m, Ischemia-reperfusion; n, myocardial ischemia/reperfusion; o, vascular endothelial cell growth factor; p, fibroblast growth factor-2; q, platelet response factor-1; r, coxsackievirus B3; s, experimental autoimmune myocarditis; t, creatine kinase; u, creatine kinase isoenzyme; v, catalase.

## Glossary

AF: atrial fibrillation
AMPK: AMP-activated protein kinase
AP-1: activator protein-1
Apo^−/−^E: apolipoprotein E gene knockout
AS: atherosclerosis
BBR: berberine
CaM: calmodulin
CHF: chronic heart failure
COX-2: cyclooxygenase-2
CVB3: coxsackievirus B3
CVD: cardiovascular diseases
CXCR4: CXC chemokine receptor 4
DCM: diabetic cardiomyopathy
DOX: doxorubicin
EAM: experimental autoimmune myocarditis
ECM: extracellular matrix
EMMPRIN: extracellular matrix metalloproteinase inducer
ERK: extracellular signal-regulated kinases
ERS: endoplasmic reticulum stress
HF: heart failure
HDL-C: high-density lipoprotein cholesterol
HIIT: high-intensity interval training
HNF-4α: hepatocyte nuclear factor 4α
HUVECs: human umbilical vein endothelial cells
IHD: ischemic heart disease
IL: interleukin
IR: ischemia-reperfusion
JAK-2: janus kinase-2
JNK: jun n- terminal kinases
LDL-C: low density lipoprotein cholesterol
LDLR: LDL receptor
LOX1: lipoprotein receptor-1
LPS: endotoxin
MAPK: mitogen-activated protein kinase
MDA: malondialdehyde
MI: myocardial infarction
MI/R: myocardial ischemia/reperfusion
MLC: myosin light chain
MMP: matrix metalloproteinase
mTOR: mammalian target of rapamycin
MyD88: myeloid differentiation factor 88
NF-κB: nuclear factor-κB
NH: neointimal hyperplasia
NO: nitric oxide
OLZ: olanzapine
ox-LDL: oxidized low-density lipoprotein
PA: palmitate
PAH: pulmonary arterial hypertension
Pak1: p21-activated kinase 1
PCI: percutaneous coronary intervention
PCSK9: proprotein convertase subtilisin/kexin9 type
PDI: protein disulfide bond isomerase
PINK1: putative kinase 1
ROS: reactive oxygen species
SHR: spontaneously hypertensive rats
SIRT1: silent information regulator 1
Sirt3: sirtuin 3
SREBP: sterol regulatory element binding protein
STAT: signal transducer and activator of transcription
TC: total cholesterol
TG: triglyceride
TLR4: toll-like receptor 4
TNF-α: tumor necrosis factor α
TRPV4: transient receptor potential vanilloid 4
Trx: thioredoxin
u-PA: urokinase-type plasminogen activator
VPCs: ventricular premature contractions
VSMC: vascular smooth muscle cells
VT: ventricular tachycardia
